# The campus microbiome: insights into soil bacterial diversity from 16S V3–V4 rRNA amplicon sequencing

**DOI:** 10.1128/mra.01293-25

**Published:** 2026-04-15

**Authors:** Jayden Nguyen, Jacob Nguyen, Tuyen Chau, Rebecca Murphy, Jarret Richardson, Ellyn Evans, George Tiller

**Affiliations:** 1 Centenary College of Louisiana 7326, Shreveport, Louisiana, USA; California State University San Marcos, San Marcos, California, USA

**Keywords:** microbiome, 16S RNA, amplicon, DNA sequencing, prokaryotes, soil microbiology, Louisiana

## Abstract

This study consisted of characterizing soil bacterial diversity via 16S V3–V4 amplicon sequencing on CCLA’s campus, with a focus on analyzing samples collected near college dormitories. The data provides a sufficient starting point for us to characterize bacterial diversity both on and off campus in Northwest Louisiana.

## ANNOUNCEMENT

To better understand soil bacterial diversity, we acquired soil samples surrounding dormitories at Centenary College of Louisiana using 16S rRNA V3–V4 amplicon sequencing. We collected soil samples within 10 to 15 cm of college dormitory air conditioning units (Table 1). The soil samples were obtained in triplicate by digging 7.62 cm into the topsoil using a Sterileware sampling spatula (Millipore-Sigma, cat. no. BAH369300000). Samples were stored in separate sterile Whirl-Pak bags (cat. no. S-22729). Considering our local climate and industrial impacts on our environment, we sought to better understand the soil bacteria diversity on campus near dormitories at CCLA using 16S rRNA V3–V4 amplicon sequencing. DNA was extracted via the ZymoBIOMICS DNA Miniprep Kit per manufacturer’s instructions (cat. no. D4300).

**TABLE 1:** Location and environmental conditions for the 16S V3-V4 amplicon sequencing soil samples

Dormitories	Municipality	Coordinates	Soil collection date	Soil type	Air temp. (°C)	Soil temp. (°C)	Relative humidity (%)	Raw reads
Rotary Hall	Shreveport, Louisiana, USA	32.48272° N, 93.73223° W	Aug-11-2025	Silt loam	30.2	30.7	61	91,606
Cline	Shreveport, Louisiana, USA	32.48238° N, 93.73165° W	Aug-12-2025	Silt loam	31.1	31.0	63	105,057
James	Shreveport, Louisiana, USA	32.48465° N, 93.73206° W	Aug-13-2025	Silt loam	31.2	34.3	69	103,754
Sexton	Shreveport, Louisiana, USA	32.48475° N, 93.73267° W	Aug-13-2025	Silt loam	31.2	34.3	69	102,124
Hardin	Shreveport, Louisiana, USA	32.48474° N, 93.73165° W	Aug-13-2025	Silt loam	31.2	34.3	69	94,460
Bicentennial Village	Shreveport, Louisiana, USA	32.48251° N, 93.72850° W	Aug-13-2025	Silt loam	31.2	34.3	69	102,266

All isolated DNA samples were prepared in triplicate. Each set of replicates was pooled prior to PCR/library preparation. PCR amplicons were generated using a 15 µL Phusion High-Fidelity PCR Master Mix, 0.2 μM V3–V4 341F and 806R primers (CCTAYGGGRBGCASCAG and GGACTACNNGGGTATCTAAT), and 10 ng template DNA. The PCR cycling conditions were as follows: 98°C for 1 min, 30× (90°C for 10 s, 50°C for 30 s, and 72°C for 30 s), and final extension at 72°C for 5 min. Sample libraries were prepared using the Vazyme-VAHTS DNA Prep Kit (Illumina V4) per the manufacturer’s guidelines. The NovoSeq Reagent Kit (0.05 M raw tags) was used to generate samples with a sequencing depth of 50,000 (250 bp paired-end) reads on an Illumina NovaSeq 6000 platform(1). Default parameters were used for all analyses except where otherwise noted. Sample reads were split, filtered, and merged, followed by tag filtration and chimera removal via PYTHON v3.6.13 (2), CUTADAPT v3.3 (3), FLASH v1.2.11 (4), FASTP v0.23.1 (5), and VSEARCH v2.16.0 (6), respectively. Amplicon sequence variants were denoised via DADA2 v1.37.0 (7), and taxonomy assignments were generated via QIIME2 v2025.04 (classify-sklearn algorithm) (2, 8) with a pre-trained Naïve Bayes classifier and the SILVA 138.2 annotation database (9).

**Fig 1:**
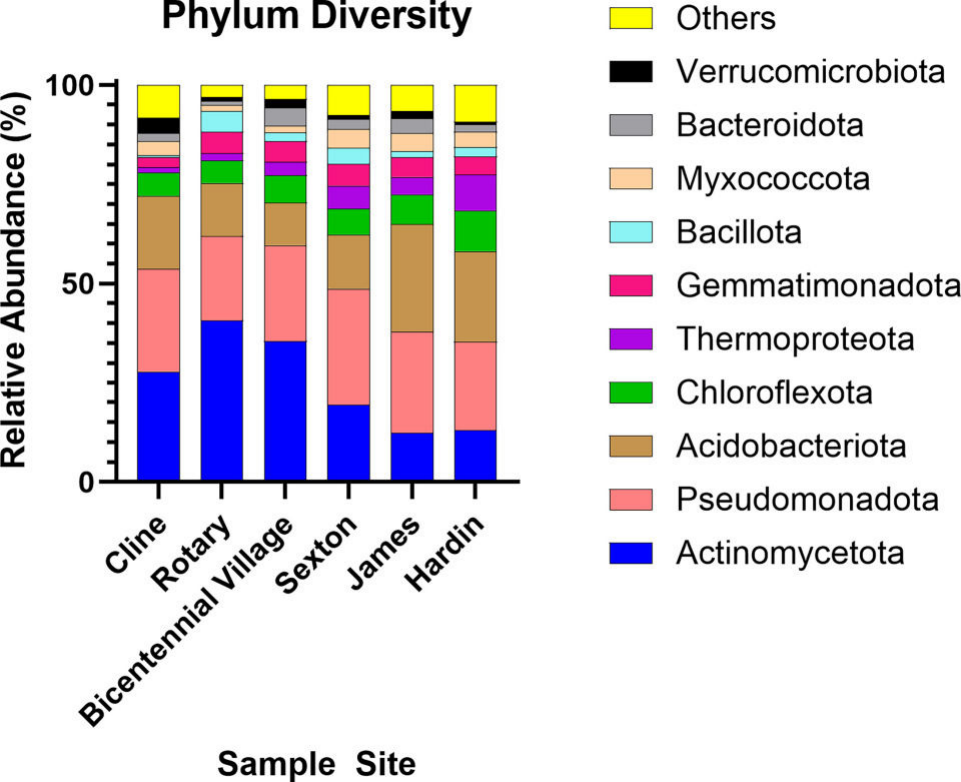
Relative abundance of prokaryotic communities at the phylum level across six dormitories. Bar plots display the relative abundance of the top 10 microbial phyla. Phyla labeled as “others” are the remaining phyla.

The relative abundance, per all samples, for the major phyla was: 12.42%–40.55% Actinomycetota, 19.38%–25.40% Pseudomonadota, and 10.65%–13.67% Acidobacteriota (Fig. 1). Future studies will focus on further characterization of soil bacteria across campus to better understand bacterial community diversity.

## Data Availability

The raw data are in the National Center for Biotechnology Information under BioProject accession number PRJNA1336157. Sequence Read Archive numbers are as follows: SRX30711479, SRX30711478, SRX3071147, SRX30711476, SRX30711475, and SRX30711474.
